# The linkage of NF-κB signaling pathway-associated long non-coding RNAs with tumor microenvironment and prognosis in cervical cancer

**DOI:** 10.1186/s12920-023-01605-9

**Published:** 2023-07-17

**Authors:** Xue Feng, Ru Shan, Xiaomeng Hu

**Affiliations:** 1 Department of Reproductive Medicine, The First Affiliated Hospital of Harbin Medical University, Harbin, 150010 China; 2 Department of Medical Psychology, Harbin Medical University, Harbin, 150010 China

**Keywords:** Cervical cancer, NF-κB signaling, Tumor microenvironment, Immunotherapy, Risk model, Long non-coding RNAs

## Abstract

**Background:**

NF-κB signaling pathway participate closely in regulating inflammation and immune response in many cancers. Long non-coding RNAs (lncRNAs) associated with NF-κB signaling have not been characterized in cervical cancer. This study revealed the linkage between tumor microenvironment and NF-κB signaling-associated lncRNAs in cervical cancer.

**Materials and methods:**

The expression profiles of cervical cancer samples from The Cancer Genome Atlas (TCGA) database were downloaded. NF-κB signaling-associated lncRNAs were screened as a basis to perform molecular subtyping. Immune cell infiltration was assessed by ESTIMATE, Microenvironment Cell Populations (MCP)-counter and single sample gene set enrichment analysis (ssGSEA). The key NF-κB signaling-associated lncRNAs were identified by univariate analysis, least absolute shrinkage and selection operator, and stepAIC.

**Results:**

Three molecular subtypes or clusters (cluster 3, cluster 2, and cluster 1) were categorized based on 27 prognostic NF-κB signaling-associated lncRNAs. Cluster 2 had the worst prognosis, highest immune infiltration, as well as the highest expression of most of immune checkpoints. Three clusters showed different sensitivities to immunotherapy and chemotherapy. Six key NF-κB signaling-associated lncRNAs were screened to establish a six-lncRNA risk model for predicting cervical cancer prognosis.

**Conclusions:**

NF-κB signaling-associated lncRNAs played an important role in regulating immune microenvironment. The subtyping based on NF-κB signaling-associated lncRNAs may assist in the selection of optimal treatments. The six key NF-κB signaling-associated lncRNAs could act as prognostic biomarkers in prognostic prediction for cervical cancer.

**Supplementary Information:**

The online version contains supplementary material available at 10.1186/s12920-023-01605-9.

## Introduction

Cervical cancer is the fourth diagnosed cancer type in females, contributing to 604,127 (3.1% of all cancers) new cancer cases and 341,831 (3.4% of all cancers) new cancer deaths worldwide in 2020 [1]. The main risk factor of cervical cancer is chronic infection by human papilloma virus (HPV) [2]. Tobacco smoke is also an important risk factor for invasive cervical cancer and cervical intraepithelial neoplasia of grade 3/carcinoma in situ [3]. HPV vaccines have been developed as an effective strategy for preventing HPV especially for human papillomavirus type 16 and 18. However, due to a long-time latency from HPV infection to malignancy, still a number of cervical cancer patients can develop.

The application of chemotherapy to radiation therapy (CRT) reaches a markedly improvement in disease-free and overall survival [4]. Nevertheless, CRT functions weak in the patients with late stages (stage III/IV) or lymph node metastases. Currently, various strategies such as adoptive T-cell therapy and immune checkpoint inhibition (ICI) have been developed to treat advanced cervical cancer [5, 6]. The efficiencies of immunotherapy vary greatly across individuals because of complicated tumor microenvironment (TME) [7, 8]. Therefore, it is essential to exploit efficient biomarkers for predicting the prognosis as well as the efficiency to clinical therapy in cervical cancer patients. Currently, the most widely used and studied biomarkers for cervical cancer are HPV DNA in cervical epithelial cells and p16INK4a protein and Ki-67 detected by immunohistochemistry [9]. A large number of reports have described biomarkers for cervical cancer, but studies is insufficient. Under realistic pressure, research efforts have been made to personalize cancer markers as indicators of specific cancer events [10].

Critical role of nuclear factor kappa B (NF-κB) in promoting tumor cell proliferation, inhibiting apoptosis, and triggering epithelial-mesenchymal transition (EMT), and inducing metastasis has been revealed [11]. Elevated NF-κB activity contributes to increased levels of pro-inflammatory cytokines that lead to pro-tumorigenic microenvironment. Thus, NF-κB signaling pathway is considered as a potential therapeutic target for cancer therapy [12]. The regulation of NF-κB signaling pathway has been discovered to be linked with long non-coding RNAs (lncRNAs) such as NKILA, MALAT1, and HOTAIR [13]. The exploration of lncRNAs in the regulation of NF-κB signaling accelerates the discovery of new therapeutic interventions. Consequently, it is of a great value to explore the potential lncRNAs related to NF-κB signaling pathway and cervical cancer progression. In this study, we identified different molecular subtypes based on NF-κB signaling-associated lncRNAs and the subtypes manifested different prognosis and response to immunotherapy. This study built a risk model with six key NF-κB signaling-associated lncRNAs for effectively predicting cervical cancer prognosis.

## Materials and methods

### Data acquisition and preprocessing

We accessed the expression profiles and clinical information of cervical cancer from The Cancer Genome Atlas (TCGA) database (https://portal.gdc.cancer.gov/projects/TCGA-CESC) [14] through Sangerbox platform [15]. Tumor samples were retained and samples without survival information were eliminated. ENSG was matched to Gene Symbol. The clinical information of TCGA dataset was shown in Table S1. Gene transfer format (GTF) file (v32) from GENCODE (https://www.gencodegenes.org/) was downloaded. LncRNAs and mRNAs in TCGA dataset were annotated according to the GTF file. The gene sets in NF-κB signaling pathway were downloaded from Kyoto Encyclopedia of Genes and Genomes (KEGG) database (https://www.genome.jp/kegg/) [16].

### Identification of NF-κB signaling pathway-associated lncRNAs

To identify the potential lncRNA regulators of NF-κB signaling pathway, according to previous studies, we developed an integrated pipeline [17, 18]. All the mRNAs were ranked based on their correlation with a specific lncRNA (adjusted by tumor purity calculated by ESTIMATE algorithm [19]). Gene set enrichment analysis (GSEA) in “fgsea” R package was used to investigate enrichment of genes of NF-κB signaling pathway. For all lncRNAs, enrichment score of NF-κB signaling pathway (TES) was measured. According to the permutation test framework, lncRNAs with significant TES were determined as the NF-κB signaling pathway-associated lncRNAs.

For lncRNA and mRNA expression matrix, LNC(i) = (lnc1, lnc2, …, lncn) and M(j) = (m1, m2, …, mn) were used to define lncRNA i and mRNA j within n patents, respectively. Tumor purity across n patients was defined as *P* = (p1, p2, …, pn) applying “ESTIMATE” R package. After removing the effects of tumor purity, the first-order partial correlation coefficient (PCC) was calculated between lncRNA i and mRNA j:

Rmp, Rlncp, and Rlncm referred to the Pearson correlation coefficients between mRNA j and tumor purity p, lncRNA i and tumor purity p, lncRNA i and mRNA j, respectively. Then, the P-value of PCC(ij) labeled as P(ij) was determined as follow:n was defined as the number of samples, and pnorm was the normal distribution function. For lncRNA i, the rank index (RI) of mRNA j was calculated as follows: $RI\left(ij\right)=-\mathrm{ln}(P\left(ij\right)* sign(PCC(ij)))$.

Sign function refers to an odd mathematical function to extract the signs of PCC(ij). All mRNAs were ranked and subjected to GSEA according to the descending order of RI. The genes of NF-κB signaling pathway signaling were mapped to the list of ordered genes. For lncRNA i, “fgsea” R package calculated the enrichment score (ES) and P-value (adjusted by FDR), which were integrated into a TES: $TES\left(i\right)=\left(1-2Pi\right)*sign(ESi)$.

The range of TES was from -1 to 1. The lncRNAs with |TES|> 0.99 and false discovery rate (FDR) < 0.05 were determined as NF-κB signaling pathway-associated lncRNAs (abbreviated as NF-κB-associated lncRNAs).

### Identification of molecular subtypes based on NF-κB-associated lncRNAs

The expression profiles of NF-κB-associated lncRNAs were the input in conducting unsupervised consensus clustering performed by ConsensusClusterPlus R package [20]. PAM algorithm and Spearman correlation were used as a distance for conducting 500 times of bootstraps with each one including 80% of TCGA samples. Cluster number k was between 2 and 10. Cumulation distribution function (CDF) curves and consensus matrix determined the optimal cluster number.

### Assessment of immune characteristics

ESTIMATE, Microenvironment Cell Populations (MCP) -counter, and single sample GSEA methodologies were applied to assess immune cell infiltration. ESTIMATE algorithm calculated immune score, stromal score and ESTIMATE score [19]. MCP-counter analyzed the estimated proportion of 10 immune-related cells [21]. Single sample GSEA assessed the enrichment of 22 immune-correlated cells through GSVA R package [22]. In addition, TIDE algorithm was used for immunotherapy response prediction based on T cell status and infiltration of immunosuppressive cells. Gene signatures of interferon-γ and cytolytic activity (CYT) were obtained from previous studies [23, 24].

### Establishment of a risk model based on NF-κB-associated lncRNAs

Firstly, univariate Cox regression analysis screened the lncRNAs significantly related to overall survival under *P* < 0.05 (defined as prognostic NF-κB-associated lncRNAs). TCGA dataset was randomly assigned into testing and training sets at a ratio of 1:1. Least absolute shrinkage and selection operator (Lasso) regression analysis [25] and stepwise Akaike information criterion (stepAIC) [26] were employed to decrease the number of prognostic NF-κB-associated lncRNAs and retain the key lncRNAs. The NF-κB-related risk model was determined as:

$Risk score={\sum }_{i=1}^{n}({Coef}_{i}*{Exp}_{i})$, where coefficients (coef) were obtained from Lasso, i indicates genes, and exp indicates the expression levels of genes. The performance of the risk model was evaluated using Kaplan–Meier survival analysis and receiver operation characteristic (ROC) curve analysis.

### Statistical analysis

All statistical analysis was performed in R software (v4.2.0). ANOVA was used to examine the difference among three groups. Wilcoxon test was used to examine the difference between two groups. Log-rank test was conducted in survival analysis and Cox regression analysis. *P* < 0.05 was determined to have statistical difference.

## Results

Identification of molecular subtypes based on lncRNAs associated with NF-κB signaling pathway.

The lncRNAs related to NF-κB signaling pathway were identified referring to a pipeline developed by Li et al. [17]. The process could be briefly described as following steps (see the details in the Materials and Methods). Firstly, mRNA and lncRNA expression profiles of cervical cancer samples in TCGA dataset were included. Then tumor purity was calculated for each sample and mRNAs were ranked by their correlation with lncRNAs. Next, GSEA was used to judge whether the genes of NF-κB signaling pathway signaling were enriched. Finally, TES was calculated for all lncRNAs, and the lncRNAs with TES > 0.99 and FDR < 0.05 were determined as NF-κB-associated lncRNAs. A total of 149 lncRNAs were finally screened and the GSEA results of partial lncRNAs were visualized in Figure S1.

**Fig. 1:**
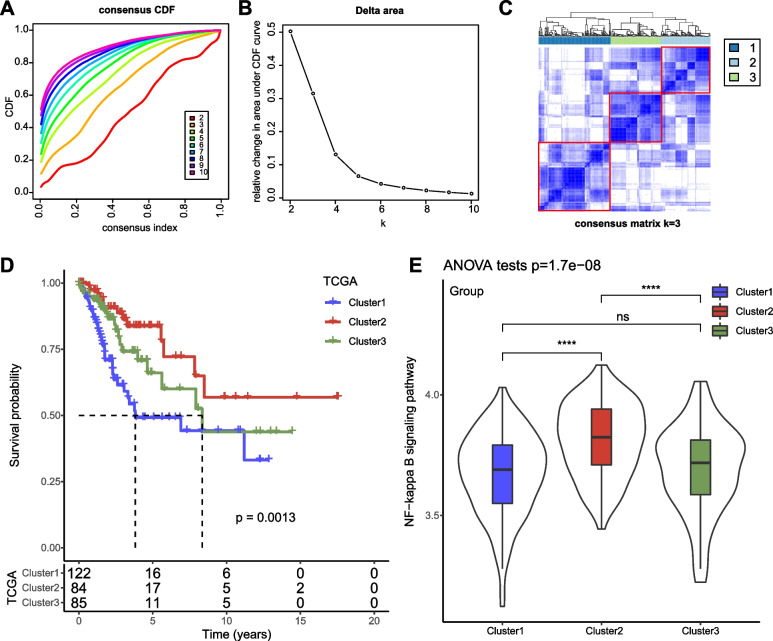
Identification of molecular subtypes based on NF-κB-associated lncRNAs in TCGA dataset. **A**-**B** CDF curves and the area under CDF curves when cluster number k = 2 to 10. **C** Consensus matrix when k = 3. **D** Kaplan–Meier survival curve of cluster 1, cluster 2, and cluster 3. Log-rank test was conducted. **E** The enrichment score of NF-κB signaling pathway of three clusters. ANOVA was conducted. *ns* not significant. ****P < 0.0001

Subsequently, we performed univariate Cox regression to identify prognosis-related lncRNAs, and found that 27 NF-κB signaling pathway-associated lncRNAs were significantly linked to the overall survival in TCGA dataset (*P* < 0.05) (Figure S2). Based on these 27 lncRNAs, we clustered samples into different clusters through unsupervised consensus clustering. Cluster number k = 3 was determined as the optimal according to CDF curve and consensus matrix (Fig. 1A-C). Ultimately, samples were assigned into three molecular subtypes or clusters (cluster 1–3). Three clusters had distinguished survival, with that cluster 1 showed the worst prognosis while cluster 2 showed the longest overall survival (*P* = 0.0013, Fig. 1D). Cluster 2 had significantly higher enrichment score of NF-κB signaling pathway than other two clusters (*P* < 0.0001, Fig. 1E), suggesting that NF-κB signaling pathway may play an oncogenic role in cervical cancer.

### Differentially expressed genes (DEGs) and clinical features for three NF-κB-associated clusters

Limma package was subjected to conduct differential expression analysis between each NF-κB-associated cluster in TCGA-CESE database and other samples in the dataset, DEGs was screened with log2(1.2) & p value < 0.05 as the threshold value. In the TCGA-CESE database, 301 DEGs existed between cluster 1 sample and the remaining sample, and 120 DEGs existed between cluster 2 sample and samples except this cluster, and 307 DEGs existed between cluster 3 samples and samples that do not belong to this cluster (Figure S3A). Only 1 of these 3 types of DEGs was identical (Figure S3B). Clinical features, including age, grade, T, N, M stage and stage did not show significant differences among the 3 NF-κB-associated clusters. The distribution of immune subtypes in the three NF-κB-associated clusters was significantly different. Although the main immune subtypes were C1 and C2, the proportion of C1 in cluster 1 was significantly higher than that in cluster 2 and cluster 3, and the proportion of C1 distributed in cluster 3 was significantly higher than that in cluster 2 (Figure S3C).

### Immune characteristics of three NF-κB-associated clusters

**Fig. 2:**
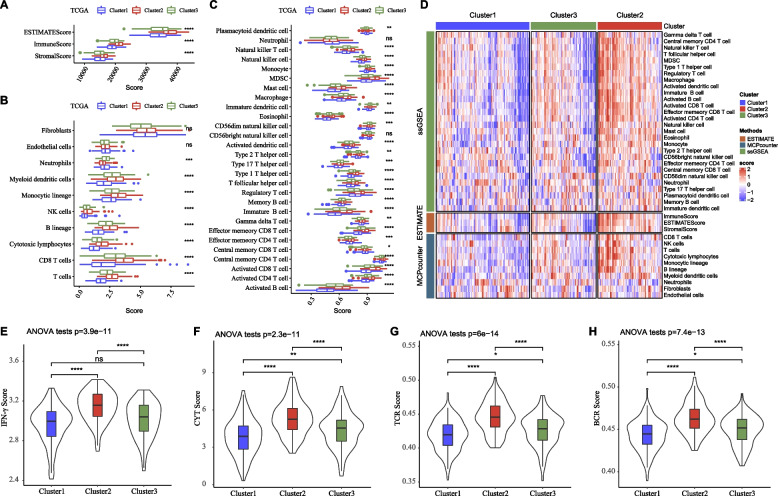
Immune characteristics of NF-κB-based three clusters in TCGA dataset. **A** Immune score and stromal score calculated by ESTIMATE. **B** The enrichment score of 10 immune-related cells calculated by MCP-counter. **C** The enrichment score of 22 immune-related cells calculated by ssGSEA. **D** The heat map of immune infiltration patterns in three clusters. **E**–**H** The scores of IFN-γ, CYT, TCR, and BCR in three clusters. ANOVA was conducted. ns, not significant. *P < 0.05, **P < 0.01, ***P < 0.001, ****P < 0.0001

**Fig. 3:**
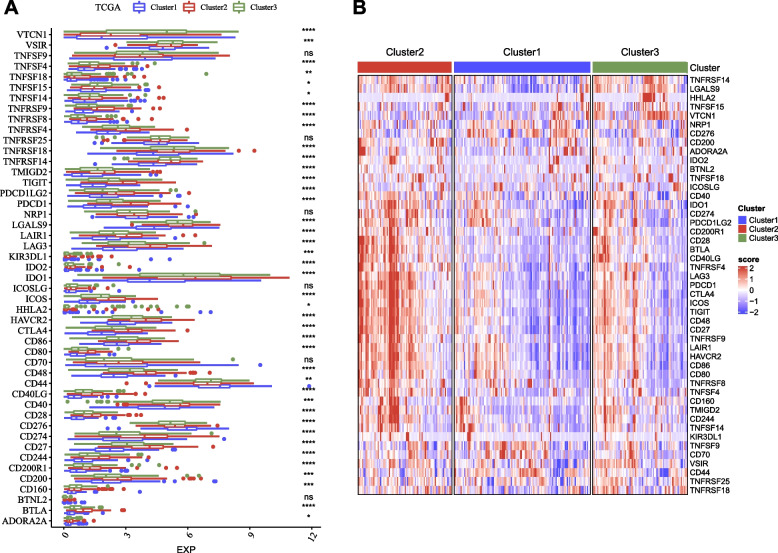
The expression of immune checkpoint genes in three clusters in TCGA dataset as shown in box plot (**A**) and heat map (**B**). ANOVA was conducted. *ns*, not significant. *P < 0.05, **P < 0.01, ***P < 0.001, ****P < 0.0001

To clarify the TME in different clusters, we applied three methodologies including ESTIMATE, MCP-counter, and ssGSEA to evaluate immune cell infiltration. ESTIMATE result showed that three clusters had different immune and stromal infiltration, where cluster 2 had the highest infiltration of immune cells and stromal cells (*P* < 0.0001, Fig. 2A, B). MCP-counter and ssGSEA revealed that most of immune cells were differently enriched in three clusters, and cluster 2 had the highest enrichment of most of immune cells, for instance, monocytes, CD4 T cells, regulatory T cells, CD8 T cells, myeloid-derived suppressor cells (MDSCs), macrophages (Fig. 2C, D). The heat map of ESTIMATE, MCP-counter, and ssGSEA results were shown in Fig. 2E. It could be evidently observed that cluster 2 was highly infiltrated of immune cells. Additionally, we evaluated the levels of IFN-γ, the scores of T cell receptor (TCR), cytolytic activity (CYT), B cell receptor (BCR) by ssGSEA. The results showed that cluster 2 had the highest scores of IFN-γ, CYT, TCR, and BCR (Fig. 2E-H), indicating that cluster 2 had a potentially activated immune response. Moreover, cluster 2 also showed the highest expression levels of most of immune checkpoint genes (Fig. 3).

### The predicted response of three NF-κB-associated clusters to immunotherapy and chemotherapy

**Fig. 4:**
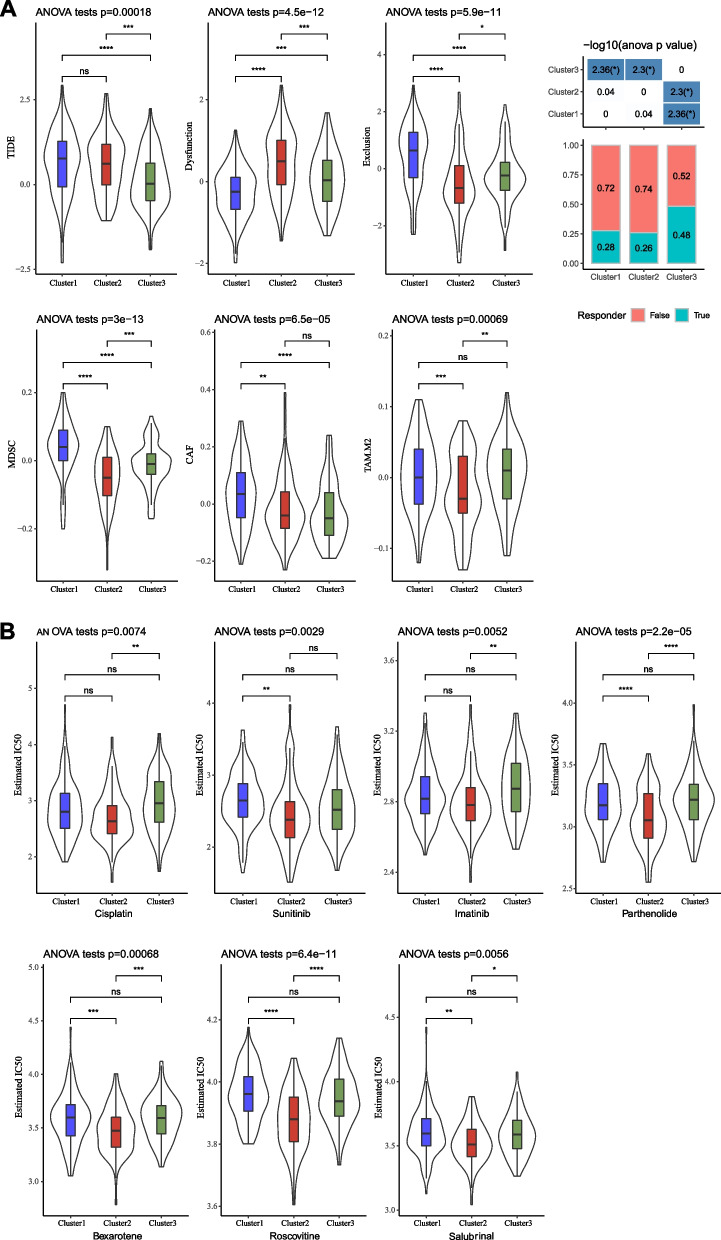
Estimating the response of three clusters to immunotherapy and chemotherapy in TCGA dataset. **A** TIDE analysis predicted T cell status, enrichment of immunosuppressive cells, and proportion of responders to immunotherapy. **B** Estimated IC50 of cisplatin, sunitinib, imatinib, parthenolide, bexarotene, roscovitine, and salubrinal. ANOVA was conducted. *ns* not significant. *P < 0.05, **P < 0.01, ***P < 0.001, ****P < 0.0001

Different TME can lead to different response to immunotherapy. In the above section, three clusters showed different immune characteristics. To find out if they had different response to immunotherapy, we employed TIDE algorithm for the evaluation. Both cluster 1 and cluster 2 showed significantly higher TIDE score than cluster 3 (Fig. 4A), indicating that they had a higher possibility to escape from immunotherapy. Although cluster 2 had high infiltration of T cells, malfunctioned T cells deterred their anti-tumor response (Fig. 4A). Cluster 1 was lacking T cell infiltration, and had the highest T cell exclusion as well as high infiltration of immunosuppressive cells including MDSC, cancer-associated fibroblasts, and M2 macrophages (Fig. 4A). TIDE analysis revealed that cluster 3 was the most responsive to immunotherapy, with an estimated proportion of 48% in positive response (Fig. 4A).

Furthermore, we assessed the potential response of three clusters to chemotherapeutic drugs by pRRophetic R package. As a result, cluster 2 showed the lowest estimated IC50 of seven chemotherapeutic drugs (cisplatin, sunitinib, imatinib, parthenolide, bexarotene, roscovitine, and salubrinal), while cluster 1 and cluster 3 showed no obvious difference (Fig. 4B). The result indicated that cluster 2 may benefit more from these seven chemotherapeutic drugs compared with other two clusters.

### Establishing a risk model based on NF-κB-associated lncRNAs

In the previous section, we identified a total of 149 NF-κB-associated lncRNAs, and we attempted to establish a risk model based on these lncRNAs. TCGA dataset was divided into training set and testing set at a ratio of 1:1. We screened a total of 10 prognostic lncRNAs in the training set through univariate Cox regression analysis (*P* < 0.05). Then we further compressed the number of lncRNAs using Lasso regression and stepAIC. Lasso regression retained 9 lncRNAs under the optimal lambda value (Figure S4A, B). StepAIC screened 6 lncRNAs as the final key prognostic lncRNAs for establishing the risk model (Figure S4C). The formula of risk model was defined as: risk score =—0.442*AC020916.1 + 0.933*AC079313.1 + 0.333*AC245128.3—0.861*AL135818.1 + 1.27*LINC02818 + 2.104*RASA2_IT1.

**Fig. 5:**
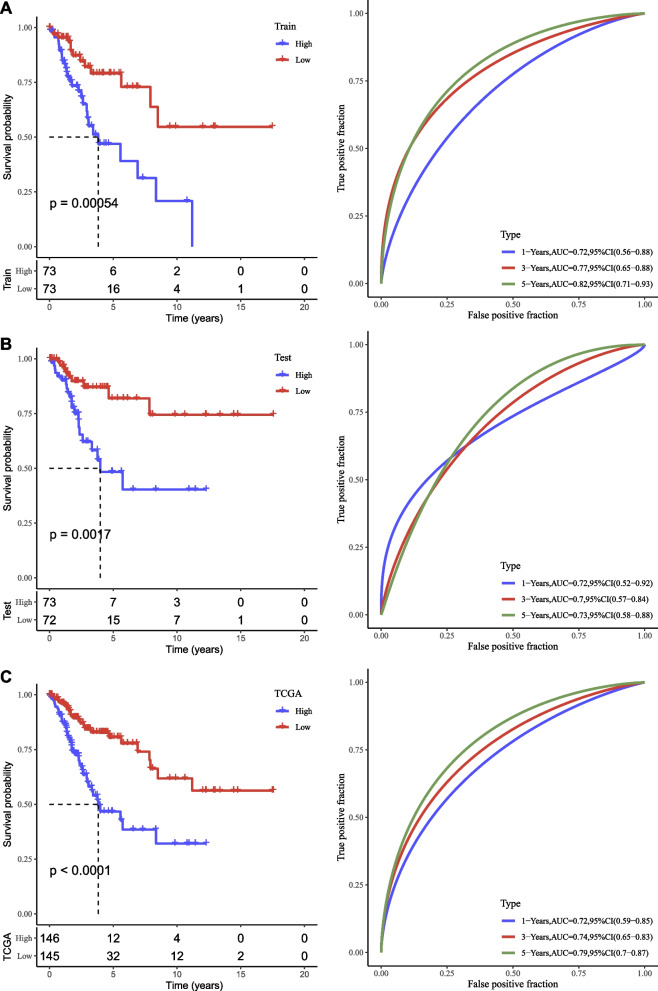
The performance of the six-lncRNA risk model in TCGA dataset. **A** Kaplan–Meier survival curve and ROC curve of the risk model in the training set. **B** Kaplan–Meier survival curve and ROC curve of the risk model in the testing set. **C** Kaplan–Meier survival curve and ROC curve of the risk model in whole TCGA dataset. Log-rank test was conducted

We validated the performance of the 6-lncRNA risk model in the testing, training sets and TCGA dataset. Risk score was calculated for each sample. Grouping of high risk and low risk was performed according to the median value as a cut-off to stratify samples. Kaplan-Meier survival curve presented that high-risk and low-risk groups had markedly different overall survival in training set, testing set and TCGA dataset (*P* = 0.00054, *P* = 0.0017, and *P* < 0.0001, respectively, Fig. 5). ROC curve analysis exhibited that the risk model was effective in predicting the survival at 1, 3, and 5 years, with AUC scores over than 0.70 (Fig. 5). Moreover, the risk model also showed a good performance in distinguishing high-risk and low-risk groups in samples with different clinical characteristics including age, T1, T2, N0, N1, M0, AJCC stage I-IV, G1 and G2 (Figure S5).

### Biological and immune characteristics of two risk groups

**Fig. 6:**
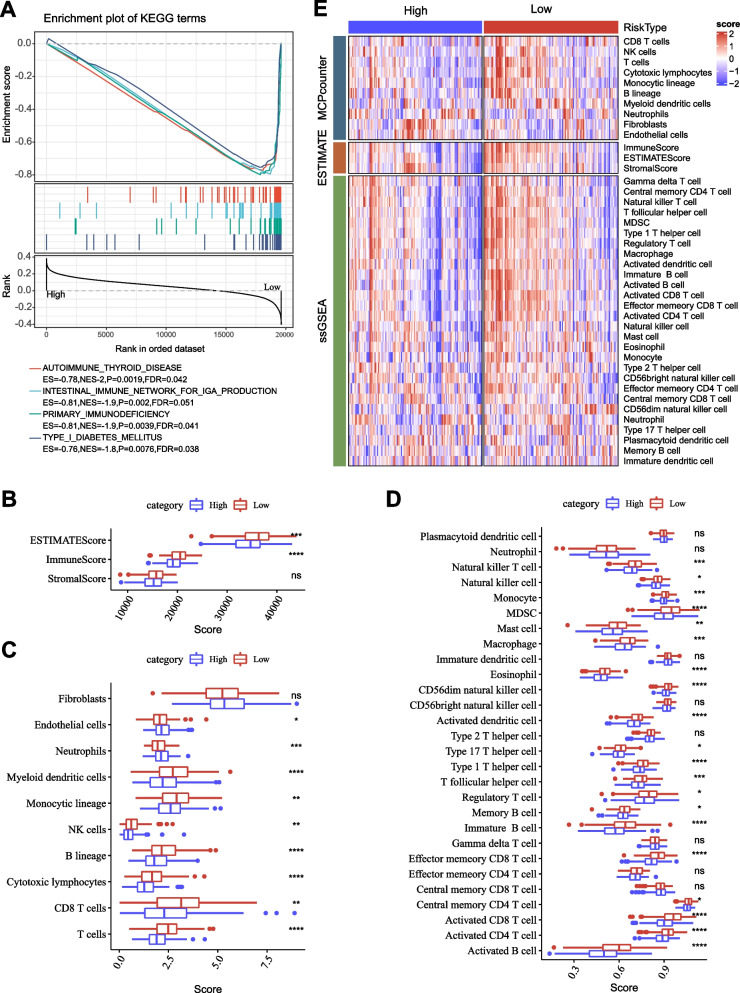
Biological pathways and immune characteristics of high-risk and low-risk groups in TCGA dataset. **A** GSEA on high-risk vs. low-risk groups. **B** Immune score and stromal score calculated by ESTIMATE. **C** The enrichment score of 10 immune-related cells calculated by MCP-counter. **D** The enrichment score of 22 immune-related cells calculated by ssGSEA. **E** The heat map of immune infiltration patterns in two risk groups. Wilcoxon test was conducted. *ns* not significant. *P < 0.05, **P < 0.01, ***P < 0.001, ****P < 0.0001

To dig out the biological difference of high-risk and low-risk groups, we used GSEA to analyze the enrichment of KEGG pathways. We found that immune-related pathways were strikingly enriched in low-risk group including autoimmune thyroid disease, intestinal immune network for IgA production, and primary immunodeficiency (*P* < 0.05 and FDR < 0.25, Fig. 6A). Immune analysis by ESTIMATE, MCP-counter, and ssGSEA indicated that low-risk group had higher immune cell infiltration than high-risk group, and most of immune cells were differently enriched in two risk groups (Fig. 6B-E). The above results suggested that two risk groups had distinguishing TME.

### Potential immunotherapy response in the two risk groups

**Fig. 7:**
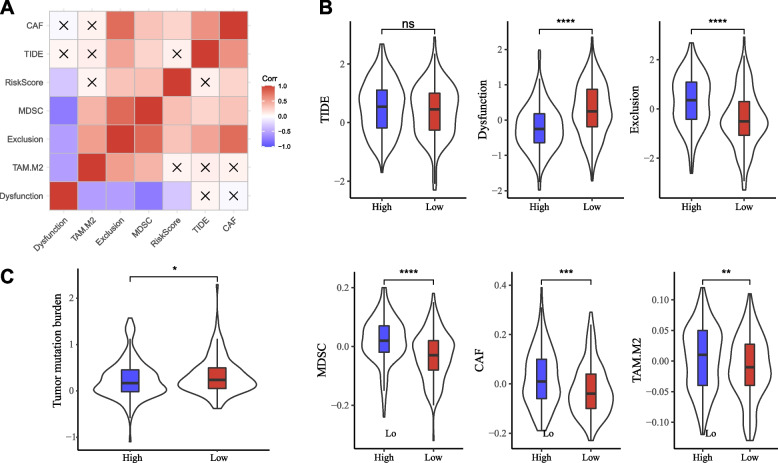
The predictive value of risk score in immunotherapy. **A** The relation of risk score with TIDE score, T cell status, and immunosuppressive cells, × sign in the square means no significant correlation. **B** The scores of TIDE, T cell dysfunction, T cell exclusion, MDSC, CAF, and M2 macrophages. **C** The relation of risk score with TMB. Wilcoxon test was conducted. *ns* not significant. **P < 0.01, ***P < 0.001, ****P < 0.0001

TIDE analysis revealed that risk score was negatively correlated with T cell dysfunction, but positively correlated with T cell exclusion, MDSC, and CAF (Fig. 7A). Two risk groups showed differences in T cell dysfunction, T cell exclusion, MDSC, CAF, and M2 macrophages (Fig. 7B). Tumor mutation burden (TMB) also has guiding significance for immunotherapy response. According to our analysis, there was a significant negative correlation between risk score and TMB, and the TMB corresponding to the low-risk group was significantly higher than that of the high-risk group (Fig. 7C). These results indicated that immunotherapy may work better at low-risk samples.The clinical value of the risk score in combination with other clinical characteristics.

**Fig. 8:**
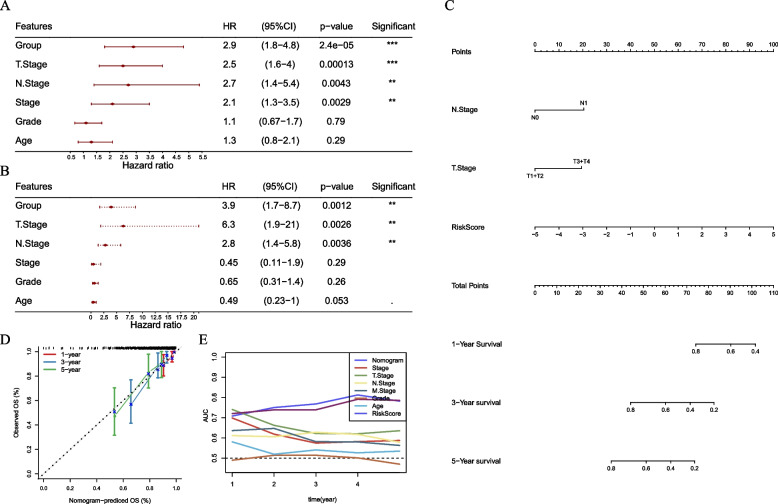
Construction of a nomogram based on risk score and clinical characteristics. (**A**-**B**) Univariate (**A**) and multivariate (**B**) Cox regression analysis of risk score, age, and clinical stages. (**C**) A nomogram based on risk score, N and T stages. (**D**) Calibration curve 1-year, 3-year and 5-year survival. (**E**) ROC curve of the nomogram, risk score and clinical characteristics

Univariate Cox regression analysis showed that risk group, T stage, N stage, and AJCC stage were risk factors while multivariate Cox regression analysis showed that only the former three were independent risk factors (Fig. 8A, B). Therefore, we included T stage, N stage and risk score to construct a nomogram for effectively predicting survival time. Risk score was shown to have the most influence to survival (Fig. 8C). Calibration curve exhibited that the predicted overall survival by nomogram was almost consistent with the actual one (Fig. 8D). Moreover, compared with other clinical characteristics, the nomogram presented the highest AUC (Fig. 8E), suggesting the highest efficiency of the nomogram in predicting prognosis for cervical cancer patients.

## Discussion

Numerous evidences have revealed the role of NF-κB signaling in inflammation, immunity, and cancer [27–29]. NF-κB signaling is suggested as a promising therapeutic target for cancer treatment. NF-κB inhibitors hinder cancer cell growth through suppressing of IκB kinase (IKK) beta activity and decreasing the nucleus translocation of NF-κB [30]. To further understand the mechanism of NF-κB signaling in cervical cancer and facilitate the exploration of NF-κB-targeted therapies, our study focused on the lncRNA regulators of NF-κB signaling and explored their influence in cancer prognosis and immune microenvironment.

We dug out a total of 149 NF-κB-associated lncRNAs through a pipeline referring to the previous study [17], and identified three molecular subtypes or clusters (cluster 1, cluster 2, and cluster 3) based on the expression patterns of NF-κB-associated lncRNAs. Cluster 2 showed the shortest overall survival and highest enrichment score of NF-κB signaling. The activation of NF-κB signaling is proved to be associated with immune cell infiltration, angiogenesis and metastasis [31], which may result in the poor prognosis of cluster 2. Cluster 1 had a better prognosis than cluster 2, but there was no significant difference on the enrichment of NF-κB signaling, suggesting the complicated role of NF-κB signaling in cancer especially in immune modulation.

Three NF-κB-associated clusters exhibited distinguished immune infiltration and proportion of different immune cells. Cluster 2 had the highest immune infiltration as well as stromal infiltration. MCP-counter and ssGSEA displayed consistent results that many immune cells were differently enriched in three clusters, where cluster 2 had the highest enrichment most of immune cells such as MDSCs, CD4 T cells, CD8 T cells, activated dendritic cells, regulatory T cells, macrophages. In addition, cluster 2 also showed the highest enrichment of IFN-γ, CYT, TCR and BCR, and these indicators are associated with active immune response. The results seemed controversial that cluster 2 had the worst prognosis but the most activated immune response. However, in addition to anti-tumor immune cells, immunosuppressive cells including regulatory T cells, M2 macrophages, and MDSCs were also highly infiltrated in cluster 2, which may lead to the attenuated immune response in cluster 2.

NF-κB has been shown to present a pro-tumor effect on macrophages and MDSCs. Macrophages are a critical group of cells in TME and they polarize to different status exerting different functions. M1 macrophages are main contributors of pro-inflammatory factors, while M2 macrophages are endowed with anti-inflammatory and immunosuppressive characteristics [32]. High infiltration of M2 macrophages and a low M1/M2 ratio are associated with poor prognosis in cancer patients [33]. NF-κB signaling is a key regulator in maintaining the function of macrophages. For example, c-Rel in NF-κB dimers is necessary for the expression of IL12B in macrophages and is important for macrophages to master inflammatory response according to transient and persistent TLR4-induced signals [34, 35]. MDSCs are considered as immunosuppressive cells and high infiltration of MDSCs is related to poor prognosis in cancer [36]. Lines of evidences have shown that NF-κB plays a supportive role in the activation of MDSCs [37, 38]. For example, MDSC function is activated by TNFR/TNFR2 signaling through an NF-κB-dependent manner [39]. The crosstalk between NF-κB and immune cells supports that these NF-κB-associated lncRNAs may serve important roles in regulating immune response in cervical cancer.

In addition, immune checkpoints are responsible for the activation of T cell function. High expression of immune checkpoints such as PD-1 and PD-L1 can suppress T cell activation [40], which is associated with poor prognosis of cancer patients [41]. We found that cluster 2 had strikingly higher expression of most of immune checkpoints such as PD-1 (PDCD1), PD-L1 (CD274), LAG3, IDO1, CD40, and CTLA-4, which are possibly responsible for the poor prognosis of cluster 2. TIDE analysis showed that cluster 3 had the lowest TIDE score and the most predicted responders to immunotherapy compared with other two clusters. In the response to chemotherapeutic drugs, cluster 2 showed higher sensitivity than other two clusters, which meant cluster 2 may benefit much from these chemotherapeutic drugs. The above results indicated that NF-κB-associated lncRNAs may serve important roles in the response to immunotherapy and chemotherapy.

Furthermore, to allow a personalized prediction for each cervical cancer patient, we identified six key NF-κB-associated lncRNAs and established a risk model based on the six lncRNAs (AC020916.1, AC079313.1, AC245128.3, AL135818.1, LINC02818, and RASA2_IT1). Among these 6 lncRNAs, AC245128.3 was found to be a prognostic necroptosis-related lncRNA for ovarian cancer [42], and AL135818.1 was found to be a prognostic necroptosis-related lncRNA for breast cancer [43], and the remaining 4 lncRNAs have not been reported to be linked with cancer biology, which need further clarification in solid experiments. Nevertheless, the six-lncRNA risk model manifested a favorable performance in predicting the prognosis of cervical cancer patients, with AUC over 0.70 of 1, 3, and 5 years. The risk score calculated by the risk model was an independent risk factor. Moreover, the nomogram based on risk score and clinical stages was superior than the risk score only.

## Conclusions

In conclusion, this study firstly identified molecular subtypes based on NF-κB-associated lncRNAs in cervical cancer. Three clusters exhibited distinct prognosis and tumor microenvironment as well as response to immunotherapy and chemotherapy. Importantly, we constructed a risk model based on six key NF-κB-associated lncRNAs that could efficiently predict the prognosis for cervical cancer patients. The six key NF-κB-associated lncRNAs may also provide a direction for the further mechanism of NF-κB in regulating immune response.

## Supplementary Information

**Additional file 1: Table S1.**

**Additional file 2: Figure S1.**

**Additional file 3: Figure S2.**

**Additional file 4: Figure S3.**

**Additional file 5: Figure S4.**

**Additional file 6: Figure S5.**

## Data Availability

All data generated or analyzed during this study are included in this published article.

## Abbreviations

BCR: B cell receptor
CRT: Chemotherapy to radiation therapy
CDF: Cumulation distribution function
CYT: Cytolytic activity
ES: Enrichment score
FDR: False discovery rate
GSEA: Gene set enrichment analysis
HPV: Human papilloma virus
ICI: Immune checkpoint inhibition
KEGG: Kyoto Encyclopedia of Genes and Genomes
Lasso: Least absolute shrinkage and selection operator
lncRNAs: Long non-coding RNAs
MCP: Microenvironment Cell Populations
MDSCs: Myeloid-derived suppressor cells
NF-κB: Nuclear factor kappa B
ROC: Receiver operation characteristic
stepAIC: Stepwise Akaike information criterion
TCR: T cell receptor
TCGA: The Cancer Genome Atlas
TES: The enrichment score of NF-κB signaling pathway
PCC: The first-order partial correlation coefficient
TME: Tumor microenvironment
