# Supplementary figures and images for: The linkage of NF-κB signaling pathway-associated long non-coding RNAs with tumor microenvironment and prognosis in cervical cancer
Source: BMC Med Genomics. 2023 Jul 17;16:169. doi: 10.1186/s12920-023-01605-9 (PMC10351132; doi:10.1186/s12920-023-01605-9)

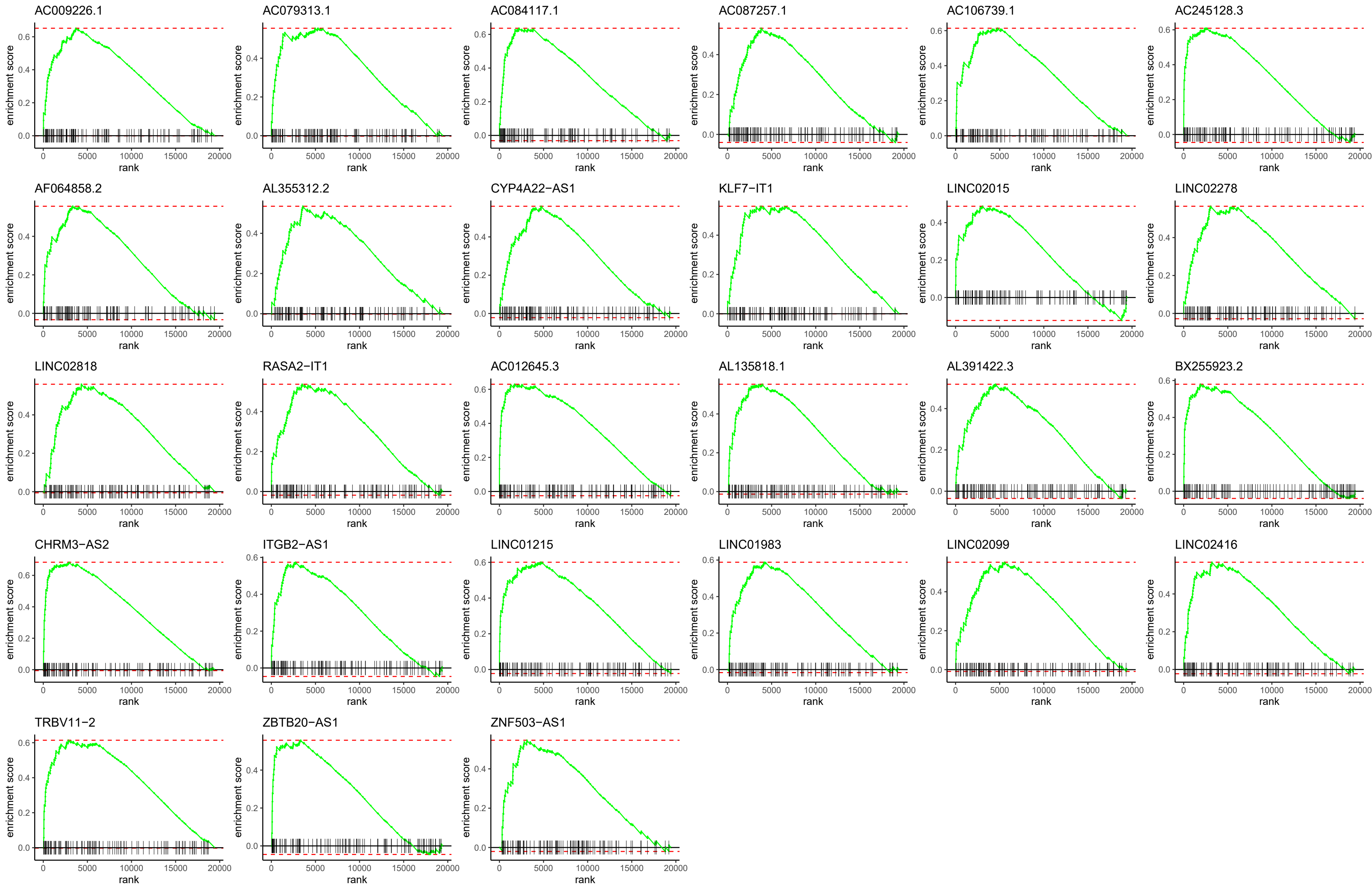

Figure S1. GSEA results of NF- $\kappa$ B-associated lncRNAs.

Supplement: Supplementary file 2 — Additional file 2: Figure S1. [file 12920_2023_1605_MOESM2_ESM.pdf]

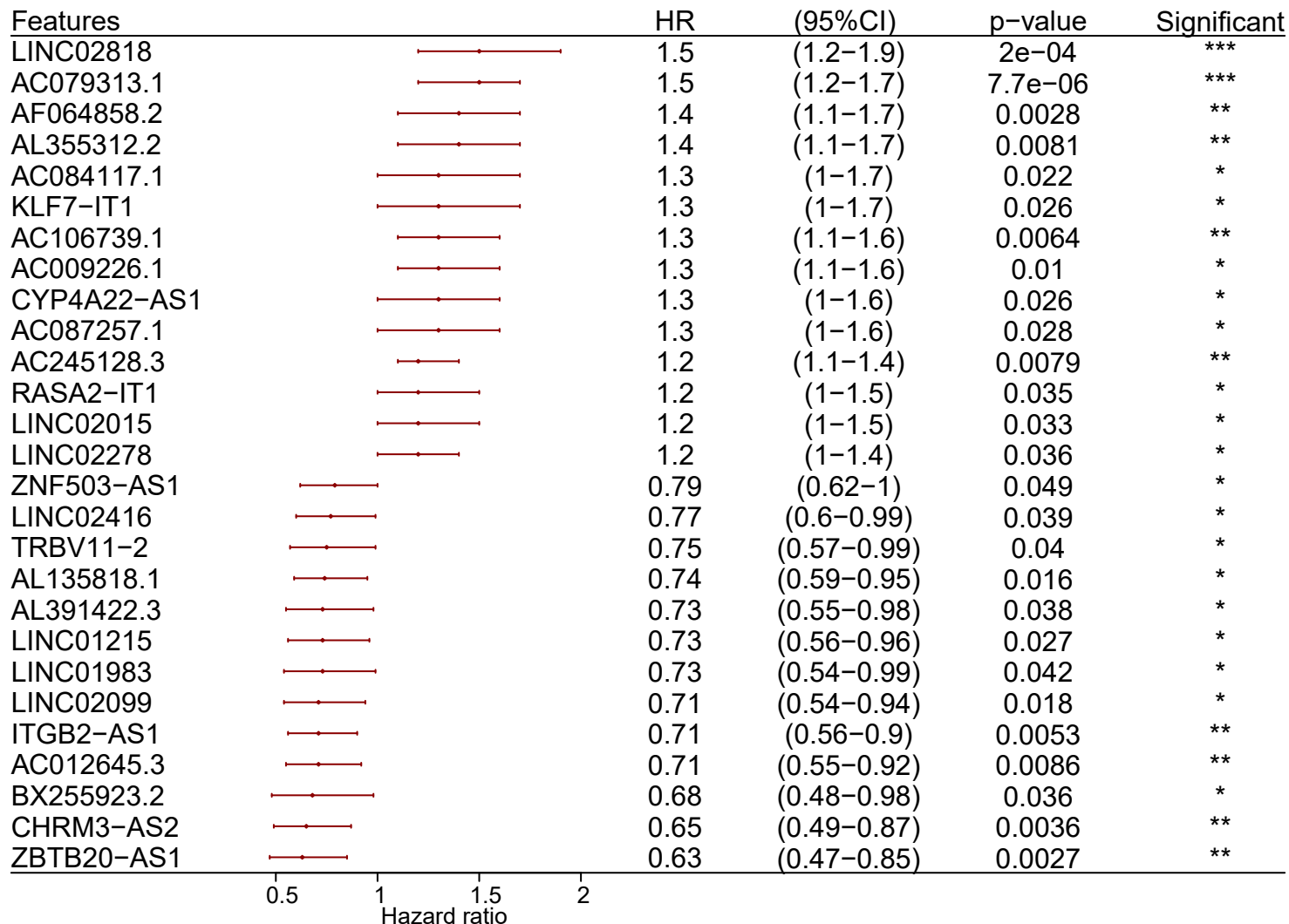

Figure S2. The forest map for the 27 NF- $\kappa$ B-associated lncRNAs.

Supplement: Supplementary file 3 — Additional file 3: Figure S2. [file 12920_2023_1605_MOESM3_ESM.pdf]
