# Supplementary material for: The linkage of NF-κB signaling pathway-associated long non-coding RNAs with tumor microenvironment and prognosis in cervical cancer
Source: BMC Med Genomics. 2023 Jul 17;16:169. doi: 10.1186/s12920-023-01605-9 (PMC10351132; doi:10.1186/s12920-023-01605-9)
Supplement: Supplementary file 4 — Additional file 4: Figure S3. [file 12920_2023_1605_MOESM4_ESM.pdf]

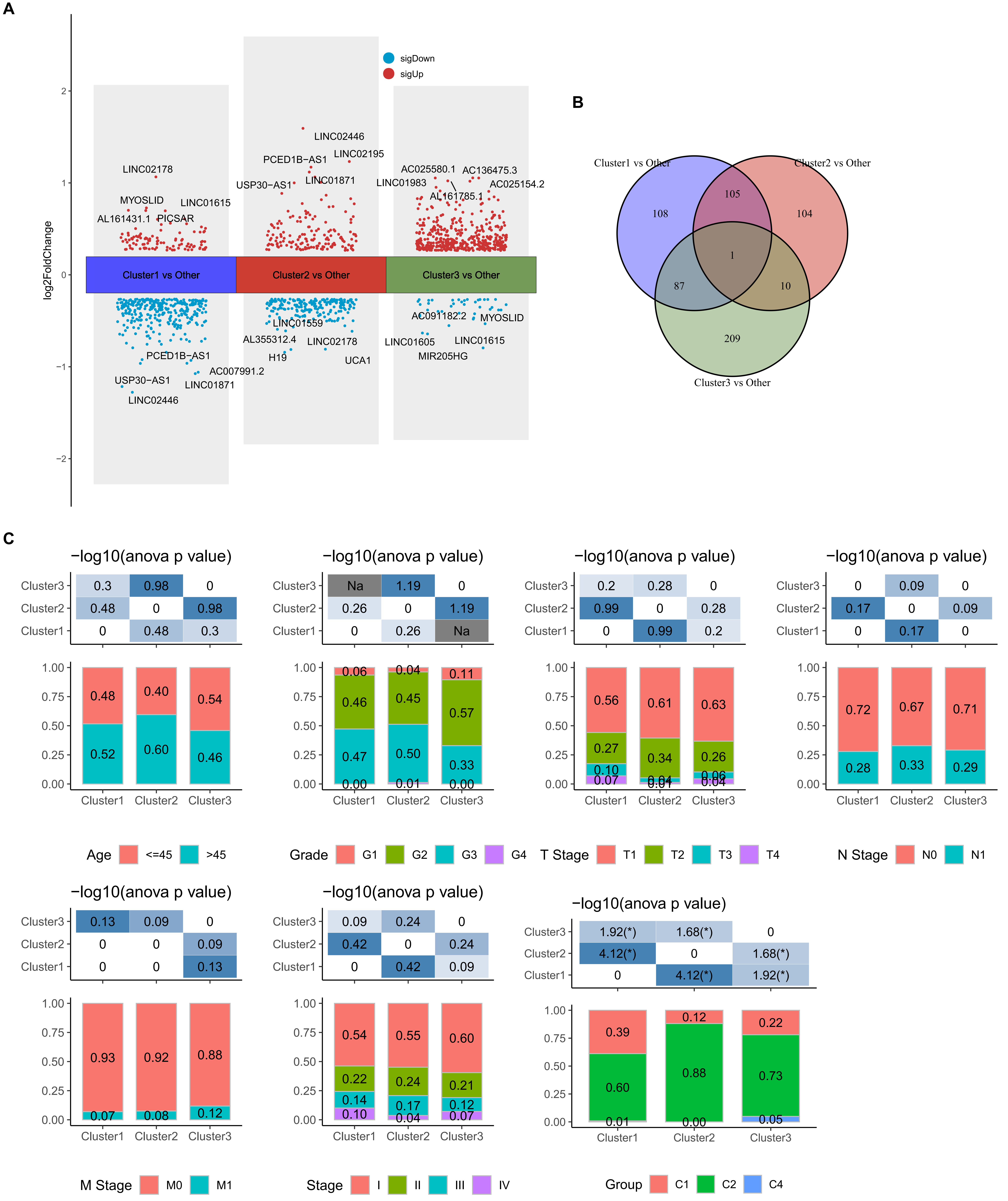


Figure S3 DEGs and clinical features for three NF-κB-associated clusters. (A) differential expression analysis between each NF-κB-associated cluster in TCGA-CESE database and other samples in the dataset. (B) Venn diagram for DEGs. (C) Clinical features difference of three NF-κB-associated clusters, including including age, grade, T, N, M stage and stage, as well as immune subtypes.
