# Supplementary material for: The linkage of NF-κB signaling pathway-associated long non-coding RNAs with tumor microenvironment and prognosis in cervical cancer
Source: BMC Med Genomics. 2023 Jul 17;16:169. doi: 10.1186/s12920-023-01605-9 (PMC10351132; doi:10.1186/s12920-023-01605-9)
Supplement: Supplementary file 5 — Additional file 5: Figure S4. [file 12920_2023_1605_MOESM5_ESM.pdf]

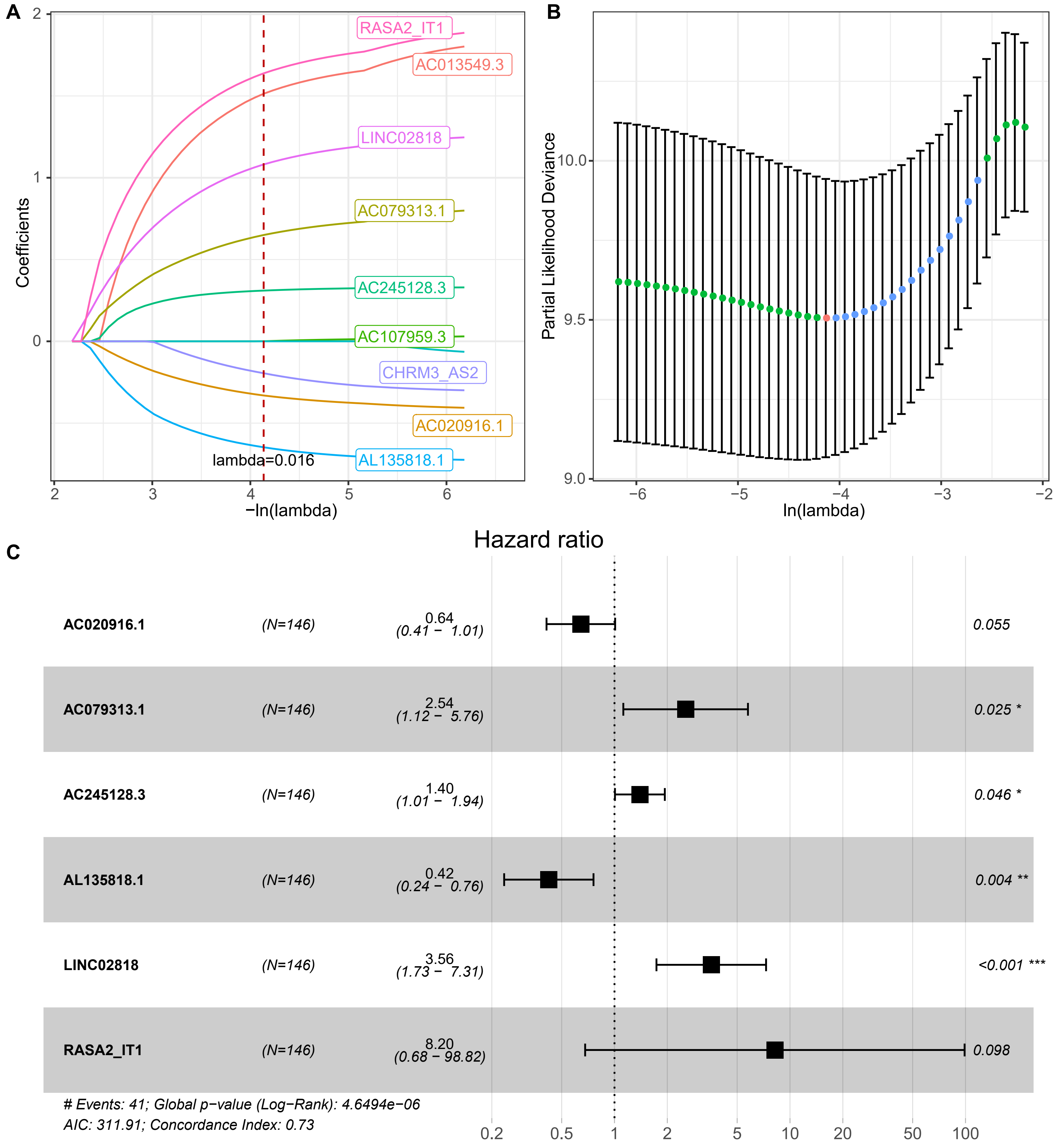

Figure S4. Establishing a risk model based on NF-κB-associated lncRNAs. (A-B) Lasso regression analysis on ten NF-κB-associated lncRNAs. Red dashed line and red dot indicate the optimal lambda value (0.016). (C) StepAIC remained the six prognostic lncRNAs. *P < 0.05, **P < 0.01, ***P < 0.001.
