# Supplementary material for: The linkage of NF-κB signaling pathway-associated long non-coding RNAs with tumor microenvironment and prognosis in cervical cancer
Source: BMC Med Genomics. 2023 Jul 17;16:169. doi: 10.1186/s12920-023-01605-9 (PMC10351132; doi:10.1186/s12920-023-01605-9)
Supplement: Supplementary file 6 — Additional file 6: Figure S5. [file 12920_2023_1605_MOESM6_ESM.pdf]

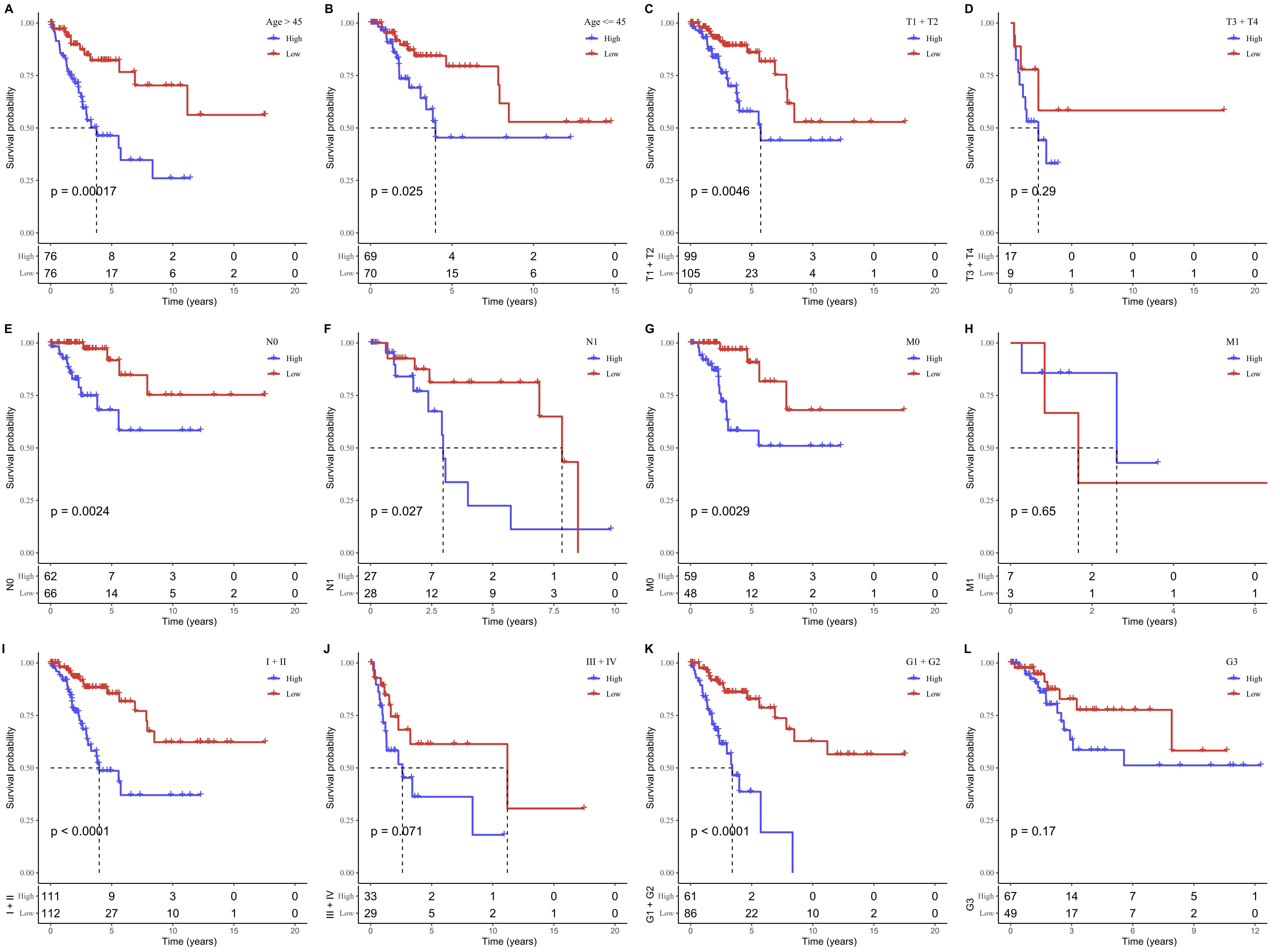

Figure S5. Kaplan-Meier survival curves of high-risk and low-risk groups in different clinical characteristics.
